# The generals and the war against COVID-19: The case of Zimbabwe

**DOI:** 10.7189/jogh.10.020388

**Published:** 2020-12

**Authors:** Noah Maulani, Israel Nyaburi Nyadera, Brian Wandekha

**Affiliations:** 1 Department of Social Policy, Ankara Yildirim Beyazit University, Ankara, Turkey; 2 Department of Government and Public Administration, University of Macau, Taipa, Macau; 3 Department of Political Science, Ankara Yildirim Beyazit University, Ankara, Turkey; 4 Department of Radio Television and Cinema, Ankara Haci Bayram University, Ankara, Turkey

On 21 March 2020, Zimbabwe’s Health Minister in a press conference announced that the first case of COVID-19 from a patient who had recently travelled from England had been recorded. This marked the beginning of rising cases of infections that reached 4893 on 12 August, 2020 and 122 deaths during the same period. Although the country has also had a significant number of recoveries totalling to 1544 as of 12 August, 2020, it is the management of the pandemic in general that is raising concern among citizens and observers alike. Already, the country was facing a series of economic and political challenges as it attempted to recover from the 2017 unanticipated coup that removed the late former president Mugabe from power after decades of his rule, followed by a disputed election that resulted into a post-election violence. Similarly, the country was yet to recuperate from the EU-US led economic sanctions on the country as well as mediocre economic policies that caused rapid depreciation and shortage of the local currency.

Zimbabwe continues to be led by individuals with military background since the 2017 *coup d’etat*. The president, the 1st vice president, minister of foreign affairs, lands minister (recently died of COVID-19), the defence minister and other executive members are all former high-ranking military officers. It is expected their experiences and training in the army would be of significant importance during the war against COVID-19. However, as the government tries to respond to the pandemic, sectors such as health, economy, education, and social services which had already been weakened by previous unrelated crises continue to show signs of vulnerability and further disruption as a result of the COVID-19 pandemic. The impact of government policies to try and curb the spread of COVID-19 have also impacted the informal sector, which provides employment to many citizens, public transport, mining, education, and agriculture [1].

This paper seeks to examine the response by the government of Zimbabwe in dealing with the COVID 19 pandemic. It argues that the training and strategies several executive members acquired from the military could be used in combating the spread of invisible enemy (Coronavirus). However, a combination of poor policies, ineffective government actions and counterproductive measures threaten to cause more harm to the Zimbabweans than the pandemic. The paper begins by providing an epistemology of the disease in Zimbabwe, then looks at how the government has responded to the pandemic. It then examines the challenges emerging from the negative impacts of the disease and the government policies on the citizens. The authors conclude with a set of policy recommendations.

## THE EXPERIENCE OF COVID-19 IN ZIMBABWE

**Figure 1:**
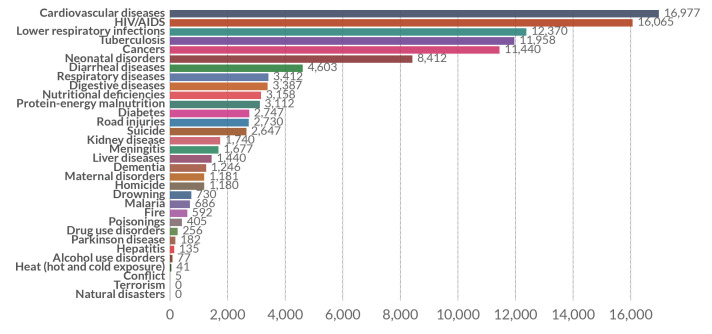
Number of deaths by cause in Zimbabwe (2017) [3].

Over three decades of economic crisis, characterised by hyper-inflation, unemployment, and corruption, overall public service delivery in Zimbabwe including the health sector has been adversely affected. Even before the outbreak of COVID-19 in Zimbabwe, the health sector was shaky, understaffed and underfinanced [2]. As of 2017 the country’s health system was struggling with a myriad of diseases (**Figure 1**) as patients died from treatable diseases such as cholera, malaria, and other cardiovascular diseases. The country has a total of 214 hospitals out of which 120 are state-run hospitals to serve a population of over 14 million people. Funding of the health sector in Zimbabwe has been affected mostly by the declining economy as well as poor policies in resource allocation. The threat of COVID-19 on health systems globally can be exemplified by the crisis faced by countries in Europe, USA, Latin America, and some Asian countries. This means the danger of a potential mass spread of the disease in Zimbabwe could spell a disaster of unprecedented magnitude. Therefore, making it even more important for policymakers, researchers, health practitioners and the citizens in general to work closely together in taming the spread of the disease.

**Figure 2:**
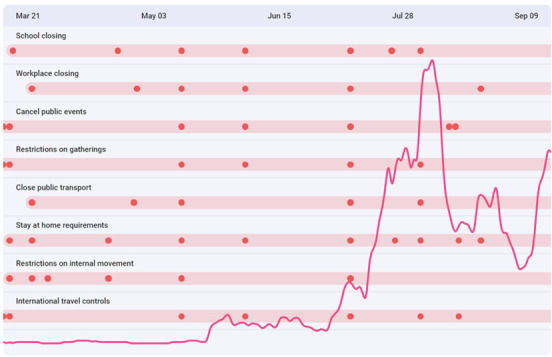
A timeline of government response and a summary of COVID-19 cases between March and September 2020 [4].

The experience Zimbabweans are going through during the pandemic is difficult one (**Figure 2**). On one hand, the uncertainty caused by the disease and government policies to curb the spread of the virus, on the other hand, is the soaring economic crisis, police brutality, abduction of political and media personalities, lack of health insurance and inadequate efforts to develop or secure a vaccine or cure for the disease. There is also a problem of food insecurity and scarcity. Over half of the country’s population (7.7 million people) are at risk of starvation should urgent humanitarian support not be effected [5]. The organisations estimate that at least US$472 million will be required to cushion the public against the food deficit in Zimbabwe.

Amid these crises where one would expect that the government to respond in a manner that instills confidence in the people, increased violent clashes and call for protests are on the rise. The government continues to insist that the country is well prepared to deal with the pandemic. According to Dr Isaac Phiri, the Deputy Director Epidemiology and Communicable Diseases the country has established over 64 isolation facilities in the cities of Harare (6) Bulawayo (3), Mashonaland West (6), Mashonaland (8) Manicaland (7) Masvingo (6), Matebeleland South (6) Matebeleland North (7) Mashonaland Central (6) and Midlands (9) [6]. Despite the establishment of these facilities, continued strikes, go-slows and boycotts by health practitioners has left many COVID-19 patients facing huge uncertainty. According to the local nurses’ union, the government has not provided frontline workers with the necessary training as well as protective gear to combat the pandemic [7]. In addition, the cost of treatment is above the reach of many citizens. Currently private hospitals are charging between US$60 and US$75 per test and over USD$3000 for general ward treatment and US$5000 for ICU treatment (Ndebele, 2020). To put the cost into context, using the current exchange rate an average teacher earns an equivalent of US$50 per month [8]. The impact of the hyperinflation has made accessing local and international currency a nightmare for many Zimbabweans. The other challenge is the lack of sufficient equipment in public hospitals that is pushing a few who can afford to go for treatment in privately owned hospitals. Apart from the inadequate bed capacity, most public hospitals lack equipment such as ventilators, oxygen machines, ICU beds and shortage of drugs.

**Figure:**
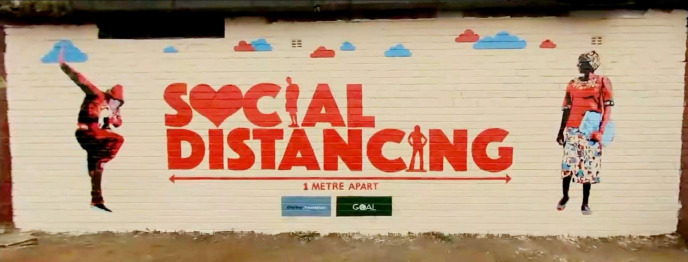
Photo: A GOAL Foundation sponsored COVID-19 sensitization picture on a wall in Mbare District, Zimbabwe (from the authors’ own collection, used with permission).

The health sector crisis is just one of many threats Zimbabweans are facing during the pandemic. Two other critical issues facing the country include police brutality and food insecurity. The latter refers to the dangerous efforts people are taking to access food even if it means crossing the border. For example, there has been sustained border crossing activities from Zimbabwe to Musina, a northern town in the Limpopo province of South Africa. People are crossing the border on foot without proper immigration documents [9]. What is concerning though is the skyrocketing number of cases of COVID-19 being registered in South Africa and which could spill over to Zimbabwe. As of 12 August 2020, South Africa had recorded 568 919 confirmed case and 11 010 deaths; and was on the 5^th^ place of countries with most cases in the world [10].

The second critical issue during the pandemic is the level of impunity and police brutality in the country. The arrest of journalist Hopewell Chin’ono, and politician Jacob Ngarivhume, who had gone public to condemn corruption particularly at the Ministry of Health marked the beginning of a planned nationwide protest against corruption on 31 July 2020 [11]. The government responded by deploying military in the capital from 28 July 2020. What followed were disturbing scenes of violence against unarmed civilians as the military and police used live ammunition. In addition to this unchecked violence, over 100 000 people have been arrested in the last four months and charged with violating COVID-19 rules [12]. Ironically, the law enforcement agents arresting people for violating COVID-19 rules are themselves not observing even the most basic rule of social distancing and wearing a mask.

The third challenge Zimbabweans are facing during this time is corruption. Corruption situation in Zimbabwe has worsened during COVID-19 pandemic. Corruption has been manifested through the lack of accountability in the use and distribution of COVID-19 funding, equipment, misreporting of donations, awarding of procurement contracts of personal protective equipment (PPEs) and medicine without following the proper procurement channel. For instance, the Minister of Health and Child Care Dr Obadiah Moyo was fired and is currently facing prosecution for awarding procurement contracts for PPEs and medicine worth US$60 million at inflated price [13]. Dr Moyo is accused of recommending a company to supply government masks at a cost of US$28 each while globally similar quality masks could be bought at less than US$1. Meanwhile, essential workers are reporting to work without proper protective equipment. The former minister has since been released on a cash bail (Z$50 000, US$2000). Corruption continues to be a disaster during COVID-19 pandemic and is hindering efforts to halt the spread of COVID-19 virus.

The fourth concern has been whether the Zimbabwean government is able to conduct sufficient number of tests. There are just few diseases that have pushed governments to encourage mass testing in this century. Apart from HIV/Aids, Cancer and TB the other disease that has led to calls for mass testing, COVID-19 is the latest in this category. The importance of testing cannot be emphasised enough as information on the number of positive cases will not only help in policy formulation, but also contact tracing and putting potential patients under surveillance. Fears of possible cases going undetected in Zimbabwe is high as the government had only managed to test 369 people in the first 40 days after the first patient was confirmed [14]. This report came on the backdrop of concerns by the Zimbabwe Association of Doctors for Human Rights, that the slow pace and high concentration of the tests in few parts of the country. Although the government has managed to test around 149 748 people, as of 9 August 2020, the ratio is still lower than other African countries [15]. The government of Zimbabwe is relying heavily on foreign aid, both financially and non-financially, for preventing, testing and containing COVID-19 cases. The number of the total COVID-19 cases is correlated with the limited number of tests conducted by the Ministry of Health and Child Care in Zimbabwe. There might be more cases of COVID-19 in Zimbabwe than the officially communicated numbers.

## GOVERNMENT RESPONSE

On 19 March 2020, the president of Zimbabwe, Emmerson Dambudzo Mnangagwa through the Statutory Instrument 76 of 2020 on Civil Protection (Declaration of State of Disaster: Rural and Urban Areas of Zimbabwe) declared COVID-19 as a national disaster. The government established a National COVID-19 Response Taskforce headed by the second Vice President Kembo Mohadi. This taskforce consists of representatives from different ministries and is further divided into subcommittees that are tasked to monitor the pandemic situation and coordinate the response to the crisis. It was also tasked with mobilising financial resources locally and internationally to cushion the country from the negative impacts of the pandemic.

On 30 March 2020, the first 21-day national lockdown was announced by the president. The terms of this lockdown included the suspension of all non-essential activities, compulsory wearing of face masks for the essentials workers and the frontline workers to wear personal protective equipment (PPEs). Statutory Instrument 83 of 2020 on Public Health (COVID-19 Prevention, Containment and Treatment) (National Lockdown) Order, 2020 gave detailed information on rules and regulations during lockdowns. The statutory instrument prohibited all forms of gatherings and restricted the movement of the public except those providing essential services. Essential services that included hospital services, state security services, emergency services such as fire brigade and ambulance services, water and electricity services, sanitary service, money transfer and exchange services, and service providers of communication were allowed to continue to operate during the pandemic.

The government established isolation centres in all 10 provinces of the country. The government ordered the closure of its borders to reduce the number of imported cases. The government closed all airports except for 3 international airports. Zimbabweans in other countries were allowed to return and it was made compulsory for them to isolate themselves for 14 days upon their return. The government allowed the movement of humanitarian workers and aid into the country. Schools were also closed, and students were asked to return to their homes until further notice.

The government allowed the usage of foreign currency (at interbank rate or ruling rate) when paying for goods and services. Recipients of remittance were allowed to withdraw money using payment agencies such as Mukuru and Western Union. This measure increased food securities in households who had access to foreign currency. However, 90 percent of Zimbabweans had no access to foreign currency as they lived paycheck to paycheck. The Statutory Instrument 87 of 2020 on Customs and Excise (Tariff) reduced the duty of fuel with the aim of enabling the essential service providers to have access to affordable fuel. The providers of transport services gained access to affordable fuel for the provision of transport to the essential workers. Statutory Instrument 88 on Customs and Excise (General) (Amendment) Regulation 101 detailed the Zimbabwe Revenue Authority (ZIMRA) may refund duty on essential goods imported for fighting COVID-19. These goods included medical supplies such as gloves, masks, and food supplies.

## LESSONS AND RECOMMENDATIONS

Since the outbreak of COVID-19 pandemic in Zimbabwe on 20 March 2020, Many Zimbabweans have suffered a double tragedy. The Zimbabwean government is led by people with military background who can be described as men of strategies. However, the way the Zimbabwean government is handling the COVID-19 response has fallen short of the expected impact on a team of former high-ranking military officials with training on strategy could do. This has left the citizens with immense uncertainty and hopelessness forcing some to call for protests, an act that can badly expose thousands more to the disease. But their desperate efforts to demonstrate against the government amid a global pandemic shows that majority of the citizens are left with limited options to turn to. The country’s economy was already devastated by a combination of international sanctions, poor governance, and economic policies as well as deep rooted corruption. The biggest challenge at this time is the fate of the citizens who have been left to seek solutions by themselves as they battle the disease, the police, and a collapsing economy. The Zimbabwean government is failing to strategize on how to reduce the spread of COVID-19 virus without inflicting more pain on its citizens who are already suffering from socio -economic constraints. However, not all is lost, there is still an opportunity for the government, the people, and other stakeholders to unite and form a formidable force that can overcome the negative impacts of the pandemic and lead to a healthy post -COVID 19 recovery. Below are some recommendations:

1. *Transparency*: Although the government of Zimbabwe has not been one of the most transparent entities, extraordinary times call for extraordinary action and this pandemic is one of such instances. The government can make available information on the number of cases, where and when the tests were taken, resources allocated to combat the disease and how they have been utilised, challenges facing the government in its responses and changing measures being implemented to keep up with the changing trends and emerging information about the disease. The government should also make public procurement processes, donations being received, efforts to make vaccine available when it is approved as well as how the government intends to cushion the citizens from the short- and long-term impacts of the disease.
2. *Effective and efficient response*: while the government has been implementing other globally popular approaches such as lock-downs, encouraging citizens to sanitize and keeping social distancing, there are issues that are particularly unique to Zimbabwe that requires the government to craft policies and adopt measure that will offer more practical solutions to the people. For example, given the already struggling economic situation in the country, the government can reach out to other development partners to help it provide face masks which are critical to reducing the spread of the disease yet very elusive especially for low-income earners [16]. The government can also reach out to the military production unit to support in the production of reusable facemasks that can be distributed to the citizens. In addition, makeshift quarantine locations, testing points as well as improve sanitation and water delivery services can be provided more in crowded urban areas and this can be done with the support of the national youth service and the military.
3. *Show leadership in addressing the growing grievances*: Amid the pandemic, the government is having to deal with growing opposition from political, professional and community organisations over different concerns. For example, the strikes called by health practitioners demanding better pay and improved working environment comes at a critical time when the government needs more personnel in dealing with the pandemic. In addressing the grievances by frontline workers, the government needs to show leadership and not its coercive powers given that some of the concerns raised by these health workers are genuine and have a bearing on whether the country can overcome the challenges of the disease. Similarly, the on -going mass action called by activists demanding accountability could have been subverted should the government responded proactively to allegations of corruption especially with COVID-19 resources.
4. *Medical cost*: there is no doubt that one of the main concerns among Zimbabweans is whether they are able to afford the medical cost for testing and treating of COVID-19. This requires serious government attention as the issue of cost for treatment is also associated with the question of accessibility and availability of sufficient medical practitioners to provide treatment to the people. To solve this problem, the government will be required to adopt a multi -dimensional approach. For example, a robust public-private partnership initiative or a memorandum of understanding where private entities can collaborate with the government or exercise social corporate responsibility and pool recourse to help subsidise the cost of treatment or testing for the citizens. Insurance companies, both public and private can include COVID-19 treatment in their most basic premium.
5. *Re-evaluate the existing strategies periodically*: While this pandemic has put many governments to test over their ability to effectively respond to public health crises, most governments seem to be trapped with few response strategies which clearly appear to be ineffective in most countries. From measuring temperatures, to claims of contact tracing, and from alleged mass testing to availability of facilities, these strategies may have worked elsewhere but their implementation in developing countries does not appear to be working. For example, contact tracing requires the use of sophisticated technology, adequate personnel, appropriate intelligence to effectively implement. Similarly, mass testing requires sufficient kits, personnel, and standby facilities where those infected can be quarantined. So far, the Zimbabwean government which claims to be using the above strategies has not proved that it has sufficient capacity to adopt these measures. Therefore, the government can re-evaluate some of these approaches and replace them with cheaper, effective, and easily adaptable approaches.
6. *Bring others on board*: Zimbabwe’s governance structure is one that can be considered to be highly centralised. This kind of governance structure has increasingly become less popular especially in the wake of emerging public administration reform measures that brought about principles such as New Public Management and Good Governance. The two approaches emphasize decentralisation not just of functions but also decision making. The current pandemic offers a unique opportunity to adopt similar measures that will see increased involvement of different actors at the national, regional and the local levels. Apart from a recommended increase in inter-governmental relations, the central and local authorities can work closely irrespective of their political affiliations to curb the spread of the disease. Local response units can be established to work closely with National COVID-19 Response Taskforce based in the capital city of Harare. These local units are able to communicate valuable information about the realities on the ground ranging from preparedness in terms of health facilities, compliance to government guidelines and other emerging issues that can hinder the effectiveness of government response. The local units will act as a source of valuable information that can be used in the formulation, readjustment and monitoring of policies and government efforts to combat the disease.

## CONCLUSION

The COVID-19 pandemic has undoubtedly disrupted different spheres of human society. In some societies, the disease continues to inflict more pain as government response measures are doing little to cushion the citizens from the pre-existing and newly emerging challenges. One such country is Zimbabwe, which amid the pandemic, the cost of living has spiked, mass protests have resulted into dangerous confrontations between the citizens and security forces, and growing uncertainty due to lack of medical insurance, increased unemployment, and declining incomes. However, not all is lost, an intelligently designed response measure, sober and responsive leadership and collaboration between the government, the citizens and the private sector could go a long way in bringing a new experience during and after the pandemic.
